# On the Use of Chloroform as a Therapeutical Agent

**Published:** 1851-03

**Authors:** James Smick

**Affiliations:** President of said Society; Indian Point, Menard Co., Ill.


THE NORTH-WESTERN
MEDICAL AND SURGICAL JOURNAL.

Vol. III.] MARCH, 1851. [No. 6

### Part I.—Original Communications.

ARTICLE I.

*On the use of Chloroform as a Therapeutical Agent;* (being a paper read before the "Central Medical Society," of Illinois,) by Dr. James Smick, President of said Society.

Gentlemen: It is made my duty, by the Constitution of this Society, to deliver an address upon some medical subject. And, in discharge of this duty, I have selected for our present consideration, the use of Chloroform as a Therapeutical Agent.

The combined experience of the medical profession is necessary to determine the dose, and select cases in which an agent of so much potency, as Chloroform, should be used. I shall, in the first place, report four cases of rare occurrence, in which I have used it with good success; and, secondly, give some rules that I have adopted to govern me relative to its use.

Case 1. On the 22d day of January, 1849, I was called to visit Miss G. I found her laboring under a very severe attack of Cerebro-Arachnitis. She had been sick some two or three days, and under the treatment of an other physician. She had been bled, purged, and blistered on the back of the neck and *forehead*, without the least mitigation of the symptoms. *Present Condition.*—Perfectly unconscious to all surrounding objects. Has high fever with restless paroxysms, which last for about ten minutes. In these paroxysms she tosses herself most violently about, requiring two or three persons to keep her in bed, and from hurting herself;—perfectly delirious, occasionally screaming vociferously. From these paroxysms she sinks into a stupor which lasts for an indefinite time, continuing sometimes as long as half an hour, at others only about five minutes, a constant muttering to herself, pulse irregular, sometimes as frequent as one hundred, and at other times down to seventy, and most of the time shattered; tongue dry and covered over with a brownish coat, breathing hard, lies with her mouth and eyes partly open when she is still,—deglutition suspended,—the surface of a purplish appearance.

Deglutition being suspended, the use of internal remedies was entirely cut off, and the aid of revulsives externally offered a poor prospect to combat so formidable a group of symptoms as this case presented. I, therefore, resolved upon the use of Chloroform to procure rest, if nothing more. I dropped thirty-five drops upon a silk handkerchief and let her breathe it until she became quiet. She lay about half an hour quite easy, she then breathed the same quantity and remained quiet for about two hours. When she roused up this time, she seemed to manifest some marks of intelligence, could talk, asked for water but could not swallow it. Ordered the Chloroform in the same quantity at about four hours intervals, if the restlessness and delirium should return; sinapisms to the spinal column about every six hours; the extremites rubbed with cayenne and vinegar, and, if she could swallow, a powder composed of 1 gr. pulv. camphor, 10 grs. calomel and 2 grs. pnlv. ipecac, every three hours, until four doses were taken.

23d. Found her much more rational than the day before; all the symptoms abated in severity, had had one operation of the bowels, had used the Chloroform but once, had made frequents attempts to take the powders but was supposed to have swallowed but one in all. The sinapisms had drawn well. Complains of pain in the head and soreness of the throat. Directed a continuation of the same remedies.

24th. The delirium seems entirely relieved, talks rationally upon all subjects, desires to take food but cannot swallow. Used the Chloroform twice since last visit, could not take the powders, had to discontinue the sinapisms on account of the irritation they produced. Has had no fever for the last twenty-four hours.

As this was the last time we had any occasion to use the Chloroform, and there was but little change in the case for three days, I think it unnecessary to give a further detail of the treatment, further than to state that every external means was used, that could be, that seemed to offer any prospect of relief in the case, such as blisters to the throat, stimulating gargles, enemata, &c. But as she could not swallow internal remedies, on the evening of the 27th a high fever set in that seemed to bring on the sinking of the vital energies of the system, and she sank on the 28th. So far as the effect of the Chloroform was concerned in this case it was perfectly satisfactory, and had deglutition returned so that she could have taken internal remedies, I firmly believe she would have recovered.

Case 2. About twelve o'clock at night on the 4th day of September, 1849, I was called to see a negro girl about sixteen years old, laboring under severe congestion of the brain. I found her on a low bed with three persons holding her there. Every ten or fifteen minutes her whole muscular system seemed violently agitated, not irregular muscular contractions, such as exist in convulsions, but more like desperate struggles to get away from the attendants. She had not spoken for about four hours, was taken in this manner at about six o'clock P. M., was perfectly insensible to external impressions ; pinching or shaking did not make any impression on her.

The pulse was rather quicker than natural, not very full nor frequent, she breathed very much like a person very tired from long exertion, no frothing at the mouth, whenever her head was at liberty she was trying to bite every thing in her way, had bitten her own arms before they held her head, did not attempt to swallow.

I took about twenty ounces of blood from her arm, and dropped about forty drops of Chloroform upon a handkerchief and applied it to her nose and mouth, but from the violent struggles that she was making a portion of it was lost.

I dropped out fifteen drops more and let her breathe it. In about two minutes she asked the attendants to let her go, raised up in bed and asked for a drink of water which she drank with great avidity, asked what time of night it was, and when told it was one o'clock she seemed astonished to think they were sitting up so late, supposed that she had just awoke from a sound sleep ; nothing but the fact that she had been bled and my presence in the room could convince her that she was indisposed.

I directed alterative doses of hyd. submuriate to be followed by a brisk cathartic. She remained rather stupid for about thirty-six hours, and then appeared to be as well as usual. I never saw any thing operate more like a charm than the Chloroform did in this case.

Case 3. I was called on the 27th of September, 1849, to visit Mrs. E., in labor with her seventh child. She had been in labor for about ten hours. The os uteri was but slightly dilated and the edges very thick and hard.

At about the sixth month of utero-gestation she received a hurt that threatened abortion ; fever followed and she was not able to be up much from that time to the present. In consequence of this indisposition she had no strength to stand a tedious labor, as this seemed likely to be. I took twelve ounces of blood from the arm and gave twenty drops of laudanum. In about eight hours I was summoned to the case again. The os uteri had dilated to its full size, the pains were very distressing, but not very efficient, her strength had evidently begun to sink, cold sweat bedewed the forehead and she was the subject of a peculiar nervous depression; while during each pain her cries were most agonizing.

Here was a case that I expected peritoneal inflammation or puerperal fever to follow, if she lived until the birth of the child. I used Chloroform, by inhalation, but not to complete anasthesia; she ceased her complaint about the pains, said she could feel them but that they did not hurt her. They increased in efficiency after the use of the Chloroform, and in about half an hour the child was born.

The placenta was delivered by the contractions of the uterus; no flooding or other pains followed. The tonic contractions of the uterus seemed to be perfect. I never saw a more rapid recovery under such unfavorable circumstances. The child also did well.

Case 4. On the second day of March last, I was called to see a child, less than two years of age, in convulsions caused by the eruptive fever of varioloid. He had two very hard convulsions, and when I got in the house he was screaming, his head thrown back, his face flushed, and his hands firmly clenched. I dropped out twelve drops of Chloroform and had him breathe it; in less than two minutes all muscular contractions were relaxed, he seemed to lay quiet for about fifteen or twenty minutes when he again became restless, when the Chloroform again quieted him. It was used some three or four times in the course of about two hours, when all symptoms of convulsions left him.

I have used Chloroform as a therapeutical agent in many other cases with marked benefit, such as neuralgia, intermittent head aches, and asthma, and, also, as an anesthetic in some surgical and dental operations. Without stopping to notice the discussion now going on between the advocates of the use of Chloroform as an anasthetic in pain attending physiological action, and those who oppose it, I shall very briefly state the principles that govern me in its use.

1. I use it in all cases of pathological action in which there is severe pain arising from nervous irritation, or inflammation demanding immediate relief, and in which the usual anodynes and narcotics would not have time to operate before a dangerous result would be apprehended, and then carry its effects just so far as to relieve pain, but not to complete anasthesia.

2. In all obstetrical cases attended by that peculiar train of morbid action, such as chlorotic habit, derangement of the chylopoietic viscera and liver, in which we have reason to apprehend that the severe and long protracted labor pains would produce a bad recovery, peritoneal inflammation or puerperal fever, &c.

3. In all obstetrical cases, when turning or instrumental delivery is necessary, in which the operation is very painful or tedious.

4. In all capital surgical operations, where the pain of the operation would do injury to the nervous system.

These are the rules that I have adopted to govern me in the use of this most potent medicine, Chloroform, and which I expect to continue to follow, until this progressive age of improvement shall bring out, and settle down upon, some other and better principles. These rules seem to be perfectly safe, not liable to the objections incident to its indiscriminate use, and freed from any fatal results, for it need not be used to complete anasthesia in any case, excepting some surgical cases.

Used in this way it supplies a vacuum that has long existed, and that could not be filled by any other medicine, except it was of the same class.

It is not extravagant to hope that the time is not far distant when Chloroform will be combined with some other agent, that its properties will be so modified that it can be used with impunity much more extensively than it now is, or that much may yet be learned relative to the dose to be used. When we reflect that it required years to ascertain the proper doses of quinine, we have much to encourage us in this particular relative to Chloroform.

*Indian Point, Menard Co., Ill.*

